# Analysis of Circulating Food Antigen-Specific T-Cells in Celiac Disease and Inflammatory Bowel Disease

**DOI:** 10.3390/ijms24098153

**Published:** 2023-05-02

**Authors:** Yasmina Rodríguez-Sillke, Michael Schumann, Donata Lissner, Federica Branchi, Fabian Proft, Ulrich Steinhoff, Britta Siegmund, Rainer Glauben

**Affiliations:** 1 Department of Gastroenterology, Infectious Diseases, and Rheumatology, Campus Benjamin Franklin, Charité-University Medicine Berlin, 13125 Berlin, Germany; 2 Institute of Nutrition, University of Potsdam, 14558 Nuthetal, Germany; 3 Institute for Medical Microbiology and Hospital Hygiene, Philipps University of Marburg, 35043 Marburg, Germany

**Keywords:** antigen-specific T-cells, celiac disease, gliadin, IBD, food antigens

## Abstract

To demonstrate and analyze the specific T-cell response following barrier disruption and antigen translocation, circulating food antigen-specific effector T-cells isolated from peripheral blood were analyzed in patients suffering from celiac disease (CeD) as well as inflammatory bowel disease (IBD). We applied the antigen-reactive T-cell enrichment (ARTE) technique allowing for phenotypical and functional flow cytometric analyses of rare nutritional antigen-specific T-cells, including the celiac disease-causing gliadin (gluten). For CeD, patient groups, including treatment-refractory cases, differ significantly from healthy controls. Even symptom-free patients on a gluten-free diet were distinguishable from healthy controls, without being previously challenged with gluten. Moreover, frequency and phenotype of nutritional antigen-specific T-cells of IBD patients directly correlated to the presence of small intestinal inflammation. Specifically, the frequency of antigen specific T-cells as well as pro-inflammatory cytokines was increased in patients with active CeD or Crohn’s disease, respectively. These results suggest active small intestinal inflammation as key for the development of a peripheral food antigen-specific T-cell response in Crohn’s disease and celiac disease.

## 1. Introduction

Antigen-specific T-cells play a central role in the adaptive immune system, promoting specific acute immune responses and the formation of immunological memory. Analyzing not only frequency, but also phenotype and function of these rare cells, represents not only a critical step towards understanding the mechanisms of adaptive immunity in general, but also in determining the specific immune status of the individual patient or diagnosing infectious or auto-immune diseases. The high diversity of the T-cell receptor, which allows for recognition of billions of different antigens, leads to an extremely low frequency of T-cells, specific for a single peptide-MHC ligand. This holds true even for pathogen-specific memory compartments in the absence of acute infections, for which specific T-cell frequencies in peripheral blood are typically far below 1%, but all the more for the naive repertoire (<0.0005%) [1,2].

Among autoimmune diseases, celiac disease (CeD) represents a model disease, as it turns active once the celiac individual is exposed to dietary gluten. Central to the celiac immune response are gliadin-specific T-cells that convey the small intestinal mucosal remodeling typical for CeD. As gluten has been identified as the disease-causing antigen, elimination of gluten results in regeneration of the duodenal mucosa and consecutive wellbeing of the patient [3,4]. However, diagnosis in CeD patients who are already on a gluten-free diet (GFD) remains challenging. Under a gluten-free diet (GFD), tissue-transglutaminase (tTG) antibodies normalize and the small intestinal villus atrophy regenerates [5]. To date, a burdening re-challenge of patients to gluten is mandatory for a valid diagnosis. However, translocation of nutritional (and pathogenic) antigens due to intestinal barrier breaches is described not exclusively for CeD, but also for inflammatory bowel diseases (IBD) [6,7]. IBD, more specifically Crohn’s disease (CD) and ulcerative colitis (UC), are also characterized by a T-cell mediated, chronic inflammation of the intestine [8]. However, when compared with CeD, the specific origin of IBD is yet unknown. CD and UC differ in their inflammation pattern as well as their distribution. While in CD the configuration of inflammation is segmental and affects all layers of the intestinal wall (i.e., transmural inflammation), inflammation in UC is limited to the mucosal and submucosal gut layers and only affects the colon [9]. Although there are first studies connecting GFD to improvement of patient wellbeing [10] and even microbiota composition [11] in IBD, to date, circulating food antigen-specific T-cells have not been analyzed in these patients. Since the small intestine is the primary contact surface for food antigens and hence for the immunological response, we analyzed the specific nutritional T-cell response in the peripheral blood of patients with small intestinal Crohn’s disease (CD), celiac disease as well as ulcerative colitis (UC), respectively. To exclude the influence of a non-intestinal inflammation, rheumatoid arthritis patients (RA) were included as control.

We applied antigen-reactive T-cell enrichment (ARTE) [12] technology to determine the specific nutritional effector T-cell response in the peripheral blood. The ARTE technique is based on the stimulation of peripheral blood mononuclear cells (PBMC) with a defined antigen and the subsequent up-regulation of the activation marker CD154^+^, which is exclusively expressed on antigen-specific CD4^+^ T-cells [13]. This method permits the detection of the entire antigen-specific CD4^+^ T-cell response just by adding the antigen of choice directly to PBMC without the need of in-vitro expanding the reacting cells. The subsequent enrichment of CD154^+^ cells enables further in-depth phenotyping of this rare cell population [14]. Thus, the ARTE technique allows direct ex vivo cytometric—and hence functional—analyses of gluten-specific, but also even rarer food antigen-specific T-cells.

## 2. Results

### 2.1. Circulating Gliadin-Specific T-Cells Are Increased in Active Disease with Ileal Inflammation

**Figure 1:**
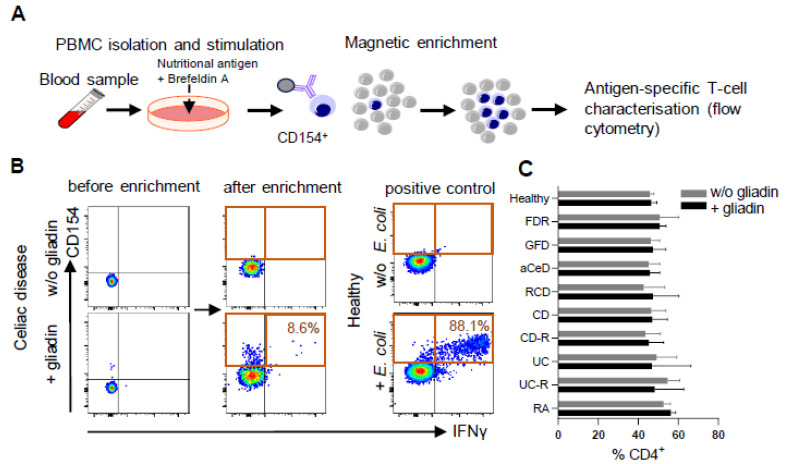
Enrichment of food antigen-specific T-cells. (**A**–**C**) Peripheral blood mononuclear cells (PBMC) were stimulated with various food antigens, magnetically enriched for CD154 and analyzed by flow cytometry. (**A**) Methodology and (**B**) exemplary density plots of CD154^+^ T-cell enrichment after stimulation with gliadin or control antigen are shown. (**C**) Frequencies of CD4^+^ T-cells in PBMC were determined from healthy controls (non-relatives, NR), patients with active Crohn’s disease (CD) or ulcerative colitis (UC), or each of these entities in remission (-R), celiac disease patients (CeD) ± gluten-free diet (GFD, aCeD) or refractory (RCD) patients, first degree relatives of CeD patients (FDR) and rheumatoid arthritis patients (RA). Data are shown as median with 95% CI.

**Figure 2:**
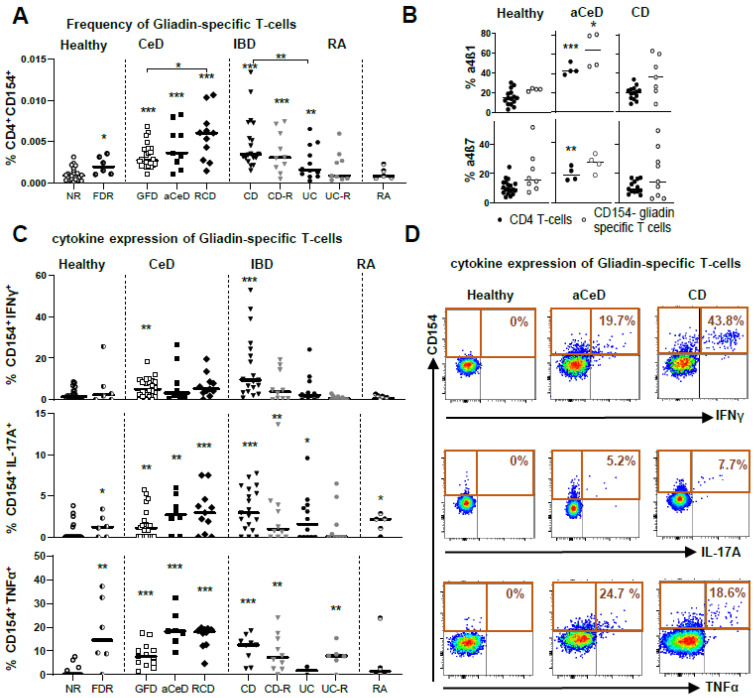
Phenotyping gliadin-specific T-cells. (**A**–**D**) Peripheral blood mononuclear cells (PBMC) were stimulated with various food antigens, magnetically enriched for CD154 and analyzed by flow cytometry. (**A**) Frequencies of CD154^+^ cells among CD4^+^ T-cells in PBMC from healthy controls (non-relatives, NR), patients with active Crohn’s disease (CD) or ulcerative colitis (UC), or each of these entities in remission (-R), celiac disease patients (CeD) ± gluten-free diet (GFD, aCeD) or refractory (RCD) patients, first degree relatives of CeD patients (FDR) and rheumatoid arthritis patients (RA) are shown. (**B**) Frequencies of CD4^+^ T cells and CD4^+^ CD154^+^ T cells, positive for integrins α4β1 and α4β7 are shown. (**C**) Frequencies of gliadin-specific IFNγ+, IL-17A+ and TNFα+ cells within CD154^+^ T-cells in between the patient groups are shown. (**D**) Exemplary dot plots of IFNγ+, IL-17A+ and TNFα^+^ CD4^+^CD154^+^ gliadin-specific T-cells are shown. Data are shown as median. Significance was determined using Mann–Whitney-U-Test. * *p* > 0.05, ** *p* > 0.01, *** *p* > 0.001. Statistically significant differences were calculated in comparison with healthy non-relatives, if not indicated otherwise.

ARTE technology was applied to all blood samples (Figure 1A) for various food antigens, including controls for antigen-specific T-cell enrichment and T-cell activation. Moreover, we clearly demonstrated the necessity for T-cell enrichment to allow for deeper cell analysis and therefore the advantage of this method over direct staining protocols for rare antigen-specific cell populations (Figure 1B). With the overall frequency of CD4^+^ T-cells remaining stable in the various disease conditions (Figure 1C), the frequency of gliadin-specific CD154^+^ T-cells among CD4^+^ T-cells in PBMC was expectedly highest in active CeD (aCeD), i.e., without GFD, as well as in refractory CeD patients (RCD). aCeD were rare patients as we did not actively initiate a gluten-re-challenge. Moreover, the frequencies were also significantly increased in CeD patients on a GFD without clinical symptoms when compared with healthy controls. Remarkably, a similar frequency to active CeD patients was observed in active CD patients with ileal inflammation (Figure 2A).

The frequency was significantly lower in CD patients in remission, in UC patients, independent of their inflammatory state and in healthy controls. Interestingly, first-degree relatives (FDR) of CeD patients, considered healthy by standard diagnostics, revealed a significant increase in the frequency of gliadin-specific T-cells compared with controls without familiar predisposition of CeD. Of notice, RA as auto-inflammatory control without intestinal inflammation, did not differ from healthy controls (Figure 2A).

Moreover, gliadin-specific CD4^+^CD154^+^ T-cells, positive for the small intestinal homing marker α4β1, but not for α4β7, a general gut homing marker, were increased in aCeD patients (Figure 2B), further strengthening the connection of peripheral nutritional antigen-specific T-cells to small intestinal inflammation.

### 2.2. Pro-Inflammatory Cytokines of Circulating Gliadin-Specific T-Cells

The subsequent functional analysis of the antigen-specific T-cells after gliadin stimulation (Figure 2C,D; Supplementary Figure S2) revealed highest production of the pro-inflammatory cytokines IFNγ, IL-17A and TNFα in cells from aCeD, Refr, and from CD patients with small intestinal involvement. Remarkably, antigen-specific T-cells of first-degree relatives of CeD patients (FDR) presented with higher frequencies of gliadin-specific T-cells and an increased TNFα expression, and were thus comparable to aCeD patients. TNFα-positive CD154^+^ cells were most discriminative when comparing active and inactive CeD to healthy controls.

### 2.3. Antigen-Specific Cells for Other Food Antigens Are Also Present in Increased Numbers in Active Disease with Ileal Inflammation

**Figure 3:**
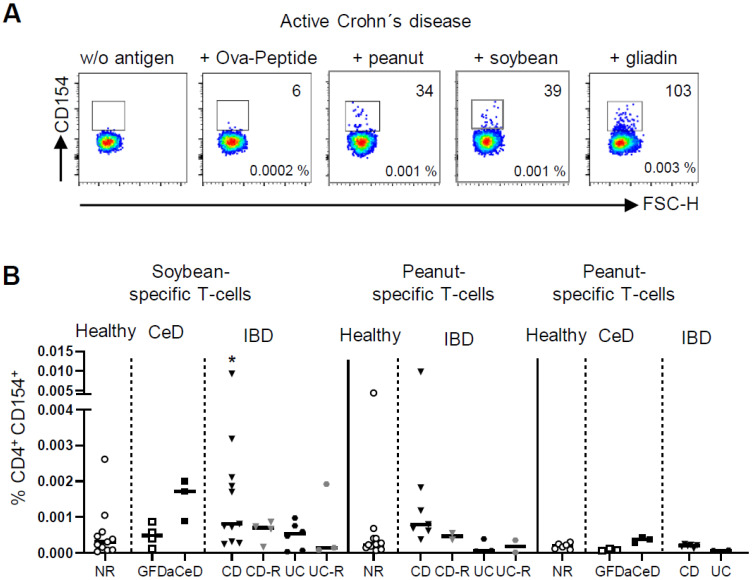
Frequencies of food antigen-specific T-cells. (**A**,**B**) Peripheral blood mononuclear cells (PBMC) were stimulated with various food antigens, magnetically enriched for CD154 and analyzed by flow cytometry. (**A**) Exemplary dot plots of CD154^+^ T-cell enrichment of indicated nutritional antigens with absolute numbers and frequencies are shown. (**B**) Frequencies of CD154^+^ cells among CD4^+^ T-cells after stimulation with soybean, peanut and OVA-peptide from healthy controls (non-relatives, NR), patients with active Crohn’s disease (CD) or ulcerative colitis (UC), or each of these entities in remission (-R), celiac disease patients (CeD) ± gluten-free diet (GFD, aCeD) or refractory (RCD) patients are shown. Data are shown as median. Significance was determined using Mann–Whitney-U-Test. * *p* > 0.05,. Statistically significant differences were calculated in comparison with healthy non-relatives, if not indicated otherwise.

To dissect a sole barrier defect, as it is present in small intestinal CD, from the disease-driving gliadin-reactivity in CeD, we included soybean protein, peanut protein and OVA-peptide in our analysis. In fact, an increased frequency of antigen-specific CD4^+^ T-cells was exclusively observed in the presence of small intestinal inflammation, namely CD and aCeD. CD patients in remission as well as UC patients independent of their inflammatory state did not differ from healthy controls. Furthermore, CeD patients on GFD, although being highly reactive to gliadin, showed no reaction to other nutritional antigens (Figure 3A,B).

## 3. Discussion

So far, the published data for ARTE have focused on bacteria- or fungi-specific antigens as well as house dust mites [15,16,17,18,19,20]. However, this method also allows the study of even rarer food antigen-specific T-cells without in-vitro expansion of the reacting cells and without re-challenging the patients. Therefore, we applied this method to detect rare food antigen-specific T-cells in peripheral blood, in order to analyze antigen reactivity for different clinical subgroups of CeD and IBD patients.

Recently, peripheral gluten-specific CD4^+^ T-cells were analyzed applying HLA-DQ2:gluten tetramers, thus identifying an increase in gluten-specific CD4^+^ T-cells in aCeD [21,22,23,24,25]. However, ARTE, as it was applied in this study, allows for a deeper analysis of the respective specific CD4^+^ T-cells to distinguish different disease states of CeD. To establish diagnosis of CeD in patients who already follow a GFD is challenging, since tTG antibodies under GFD normalize and small intestinal villous atrophy regenerates. This clinical need is growing, given the increasingly popular gluten-free lifestyle in the western world [26,27], or for first degree relatives, who frequently initiate a GFD when a household member is diagnosed with CeD. For the latter, the high risk of developing CeD has been proven in many studies [28,29] and surveillance for CeD is even recommended for first-degree relatives of a diagnosed patient where carriage of a risk gene has not been excluded [30,31]. Work herein might be the first step towards identifying such cases, without a conventional burdening gluten re-challenge, since characteristic changes in cytokine expression in gliadin-specific CD4^+^ T-cells in the peripheral blood are present. For the rare subgroup of RCD patients, especially for type I, the specific immunological nature remains unclear. Diagnosis is still based on histopathology alone, while recent studies suggest a heterogeneous composition of different pathologies to be merged under this term. In this respect, the ARTE technique for gliadin-specific T-cells represents a unique research tool for future studies that has the potential to contribute to a subclassification of this disease group. Furthermore, our data reveal a specific immunological phenotype of gliadin-specific CD4^+^ T-cells in FDR to CeD regarding a hypersensitivity towards gluten, even if diagnosed as healthy, based on their tTG status. It is well-known that FDR harbor a higher genetic risk for developing CeD. As such, it has been shown that FDR reveal an increased intestinal permeability compared with healthy controls CeD [29,32,33]. Composing our data and previously published data on permeability and disease risk to a single picture, one has to emphasize further the necessity to screen FDRs for CeD development, as is already pointed out in various clinical guidelines. By demonstrating an active immune response against the pathogenic antigen, identification and even phenotyping of gluten-reactive T-cells from peripheral blood might represent an interesting alternative diagnostic modality, all the more in pediatric cases, where prevalence is higher, invasive endoscopy is meant to be avoided. Overdiagnosis should not occur to a relevant extent, if diagnostic methodology for CeD is applied and interpreted adequately. Thus, this novel approach could fulfill the clinical need for a noninvasive marker of CeD activity as a clinical and research tool [34].

With regard to IBD, which shares the characteristics of barrier disruption [7] and subsequent intestinal inflammation in the lamina propria, we detected increased levels of gliadin-specific T-cells in the peripheral blood of active CD patients with concurrent small intestinal inflammation, paralleled by the highest frequency of antigen-specific T-cells expressing pro-inflammatory cytokines. This distinct occurrence suggests small intestinal barrier disruption as a major cause for the observed T-cell activation, since these cells express small intestinal homing markers. The homing to the ileum (α4β1) is described as an essential pathway in CD [35]. Therefore, only in these three patient groups of active small intestinal inflammation, did effector-memory T-cells outnumber the naïve phenotype among gliadin-specific T-cells in the peripheral blood. Furthermore, peripheral T-cells from CD patients with small intestinal inflammation proved to be responsive to other major food antigens [36], while neither active UC, nor CD or UC in remission, showed any reaction. Again, only antigen-specific T-cells from active CeD, but not GFD patients demonstrated similar properties, corroborating on the one hand the leaky barrier of the affected small intestine as the site of food antigen translocation and subsequent T-cell activation. On the other hand, the significant effect of gliadin, but no other food antigen, in the GFD group further confirmed the singular antigen-driven nature of CeD. Nevertheless, based on surveys, it has been suggested that long-term GFD improves gastrointestinal symptoms in active IBD patients [10]. With the present study, we are able to convey cellular and functional data by demonstrating an enhanced gliadin-specific response of pro-inflammatory cytokines towards gliadin for CD patients, which is not found in UC patients. This occurs somewhat in parallel with the detection of anti-*Saccharomyces cerevisiae* antibodies (ASCAs) in CD, but not in UC [37,38], which might reflect the increased small intestinal permeability for peptide antigens found in CD but not in UC. Interestingly and in line with our study, ASCAs were also found in patients suffering from CeD, again suggesting that small intestinal antigen processing might be pivotal [39,40].

This study has a number of limitations that include the small sample size, the monocentric design, the lack of a group of very young patients/children and maybe also the lack of a group of patients suffering from colonic Crohn’s disease. Nevertheless, recent genomic data indicate that Crohn’s disease of the small intestine is distinct from Crohn’s colitis and that small intestinal CD is specifically different from UC [41,42]. Since we aimed to emphasize these very different pathologies, we decided to focus on small intestinal Crohn’s and UC. However, a more complete view on this immune pathology including Crohn’s colitis might have added the option to recognize, if the differential extent of gliadin-specific T-cells reflects mostly the distribution type of the IBD, or if it is distribution-independent and disease-specific, maybe secondary to the transmural nature of CD.

In summary, our data suggest that small intestinal inflammation is key for the development of a nutritional antigen-specific T-cell response. Therefore, ARTE allows the distinction of CD with small intestinal inflammation from UC and CD in remission by a unique profile of circulating antigen-specific T-cells, and raises the question of whether a well-defined nutritional regimen (e.g., GFD) might have therapeutic potential in the setting of IBD. Hence, based on the analysis of the systemic immune response, an “anti-inflammatory” diet could be developed and monitored. In addition, this technique allows detailed analyses of gliadin-specific T-cells at such a high resolution that even healthy first-degree relatives can be discriminated and might thus provide a novel non-invasive diagnostic tool to identify symptom-free CeD patients on a gluten-free diet.

## 4. Methods

PBMC from CeD, CD, UC and rheumatoid arthritis patients as well as healthy controls (Table 1 and Table 2) were cultured for 6 h in the presence of defined antigens followed by magnetic enrichment of activated CD154^+^ T-cells (as marker for antigen-specific T-cells) [12] (Figure 1A,B).

### 4.1. Patients

PBMC of CD patients with small intestinal manifestation and UC patients with either active disease or remission were analyzed. Activity of disease was analyzed clinically as defined by well-established activity scores including Harvey–Bradshaw Index (HBI) and partial Mayo score (pMayo) [43,44]. Additionally, CeD patients on a GFD for at least one year, newly diagnosed active CeD patients still exposed to gluten (aCeD), or GFD-refractory CeD patients (RCD) were included. Moreover, healthy first-degree relatives to CeD patients (FDR) on a regular diet without symptoms were included. The diagnosis of CeD was based on the presence of tTG antibodies in the serum and characteristic histopathological features in duodenal biopsies (Marsh score > 1). RCD diagnosis was based on the presence of a Marsh III enteropathy and clinical malabsorption in spite of consumption of a gluten-free diet for at least one year. Clonality analysis was performed by PCR of the CDR3 region of the TCR. Detection of a clonal T-cell population and aberrant lymphocytes by immune phenotyping of duodenal tissue allowed for diagnosis of RCD type II. All other refractory cases were diagnosed as RCD type I [45]. HLA-DQ status could not be determined for IBD patients and controls. Additionally, non-intestinal inflammatory control group PBMC from rheumatoid arthritis (RA) patients were analyzed.

### 4.2. Blood Donors and PBMC Isolation

Peripheral blood samples were obtained from healthy donors and patients of the Charité-Universitätsmedizin Berlin, Medical Department, Division of Gastroenterology, Infectious Diseases and Rheumatology. All blood donors gave informed consent and the study was approved by the ethical committee of the Charité-Universitätsmedizin Berlin. PBMC were freshly isolated from 20 mL blood by density gradient centrifugation (Biocoll; Biochrom, Berlin, Germany). Heparinized whole blood was layered on the Biocoll Separation Solution and centrifuged at 1200× *g* for 25 min at 21 °C. PBMC were collected from the interphase, washed and resuspended in RPMI1640 (Gibco, Life Technologies, Darmstadt, Germany) supplemented with 5% human AB-serum (Sigma-Aldrich, St. Louis, MO, USA).

### 4.3. Antigen-Reactive T-Cell Enrichment

Identification and enrichment of antigen-reactive T-cells was performed by applying the recently described ARTE technique [12]. Briefly, 0.5–1 × 10^7^ PBMC were cultured in RPMI1640 supplemented with 5% human AB-serum and stimulated for 6 h with 1 µg/mL CD40 (Miltenyi Biotec, Bergisch Gladbach, Germany) in the presence or absence of the pepsin-trypsin digested 33-mer gliadin peptide (200 µg/mL) (Sigma-Aldrich), OVA-peptide (Invitrogen), or soybean or peanut extract (200 µg/mL) (Greer Laboratories, Lenoir, North Carolina, United States). For the last 2 h, 1 µg/mL brefeldin A (Sigma-Aldrich) was added. Cells were indirectly labeled with anti-CD154-biotin antibody, followed by anti-biotin MicroBeads (CD154 MicroBead-Kit, Miltenyi Biotec), and magnetically enriched using MS columns (Miltenyi Biotec).

### 4.4. Flow Cytometric Cell Analysis

Surface staining was performed on the MS column (first panel: Brilliant violet 510™ anti-human CD4; RPA-T4, Brilliant Violet 421™ anti-human CD197 (CCR7); G043H7, PE/Cy7 anti-human CD45RA; HI100; second panel: Brilliant violet 510™ anti-human CD4; PE/Cy7 anti-human CD29/ß1; TS2/16, PE anti-human ß7; FIB504, all from BioLegend (Koblenz, Germany); VioBlue anti-human CD49d/α4, MZ18-24A9, from Miltenyi Biotec (Bergisch Gladbach, Germany); and human FC block, from CSL Behring (Marburg, Germany)). The enriched cell fraction was fixed using eBioscience™, FoxP3 staining buffer (Thermo Fisher Scientific, Waltham, MA, U.S.A.). Intracellular staining was performed: APC anti-human IFNγ; 4S.B3, APC/Cy7 anti-human IL-17A; BL168, PerCP/Cy5.5 anti-human TNFα; MAb11, all from BioLegend; and FITC anti-human CD154 (5C8) from Miltenyi Biotec. Flow cytometry analysis was performed on an FACS Canto II device (BD Bioscience, Heidelberg, Germany). Data were analyzed with FlowJo analysis software (Ashland, OR, U.S.A.) (Supplementary Figure S1).

### 4.5. Statistics

Statistical analysis was performed using Prism software (GraphPad Software). Significance was determined using Mann–Whitney U-Test as indicated. * *p* > 0.05, ** *p* > 0.01, *** *p* > 0.001.

## Figures and Tables

**Table 1:** Patient characteristics: Celiac disease and controls.

		Non-Relative Controls (*n* = 24)	First-Degree Relatives (*n* = 6)	Celiac Disease on GFD (*n* = 24)	Active Celiac Disease (*n* = 9)	Refractory Celiac Disease (*n* = 11)
**Age** (mean ± SD)		33.3 ± 9.4	34.5 ± 9.5	43.9 ± 16.8	47.3 ± 12.4	61.1 ± 11.6
**Female** [%]		55	83	80	66	82
**tTG** (mean ± SD) [U/mL] ^#^		1.6 ± 0.7	1.4 ± 0.4	6.3 ± 4.8	114.2 ± 70.9	21.0 ± 27.2
[CE]		-	-	-	3428.1 ± 1313.7	-
**Marsh grade at first diagnosis**	IIIa IIIc IIIb	-	-	10 9 4	3 3 2	6 2 2
**RCD** type I /II [%]		-	-	-	-	63.6/36.4

GFD, gluten-free diet; tTG, tissue-transglutaminase. ^#^ standard range tTG-IgA [U/mL] < 10 U/mL; [CE] < 20 CE.

**Table 2:** Patient characteristics: Inflammatory bowel disease and controls.

	Non-Relative Controls (*n* = 24)	Rheumatoid Arthritis (*n* = 5)	Crohn’s Disease (*n* = 19 + 13)	Crohn’s Disease (Remission) (*n* = 10 + 4)	Ulcerative Colitis (*n* = 12 + 7)	Ulcerative Colitis (Remission) (*n* = 9 + 2)
**Age** (mean ± SD)	33.3 ± 9.4	49.4 ± 10.8	36.2 ± 9.3	41.4 ± 13.9	41.0 ± 14.9	42.0 ± 15.1
**Female** [%]	55	67	50	57	37	64
**Clinical score:**						
**HBI**	-	-	5.1 ± 2.7	0.5 ± 1.2	-	-
**partial Mayo**	-	-	-	-	3.9 ± 1.9	1.0 ± 1.0
**Montreal classification:**	-	-				
- A1 < 17 years			0	0	0	0
- A2 17–40 years			23	7	7	6
- A3 > 40 years			9	7	12	5
Crohn’s disease						
- L1 ileal			11	4		
- L3 ileocolonic			12	8		
- L4 upper GI			6	2		
Ulcerative colitis						
- E1 proctitis					0	0
- E2 distal UC					8	8
- E3 extensiveUC					10	3

HBI, Harvey–Bradshaw index. *n* = patients with gliadin stimulation + patients with other food antigen stimulation.

## Data Availability

Primary data will be made available upon request.

## Supplementary Materials

The following supporting information can be downloaded at: https://www.mdpi.com/article/10.3390/ijms24098153/s1.
