# Supplementary material for: Analysis of Circulating Food Antigen-Specific T-Cells in Celiac Disease and Inflammatory Bowel Disease
Source: Int J Mol Sci. 2023 May 2;24(9):8153. doi: 10.3390/ijms24098153 (PMC10179603; doi:10.3390/ijms24098153)
Supplement: Supplementary file 1 [file ijms-24-08153-s001.zip › ijms-2331596-supplementary.pdf]

Supplementary Figures

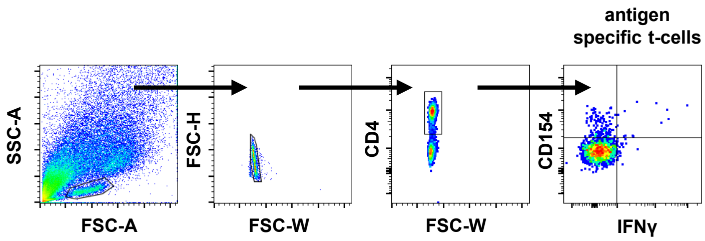

Figure S1. Gating strategy after magnetic enrichment of CD154+ (antigen specific) t cells

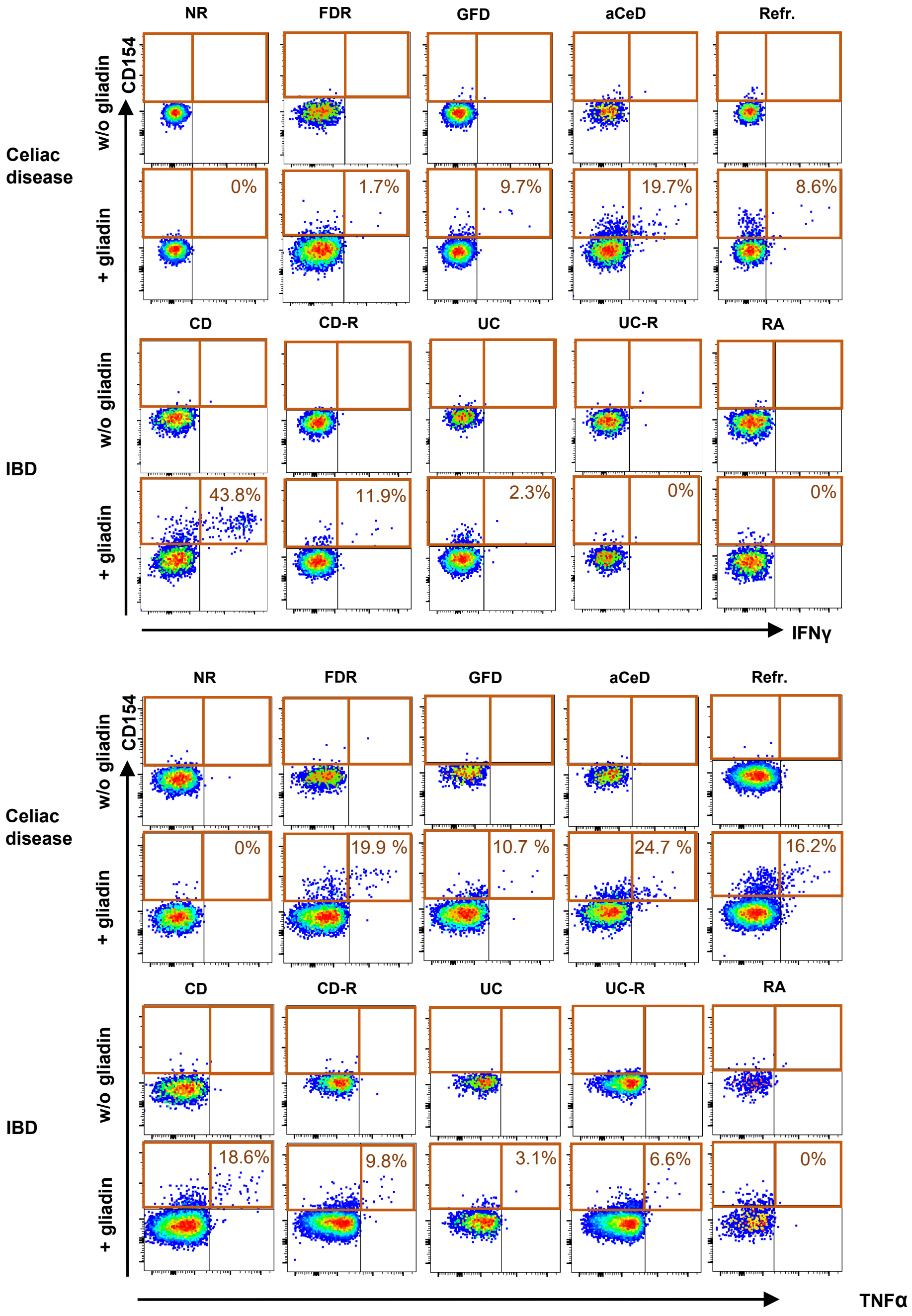

Figure S2. Cytokine frequencies of antigen (gliadin)-specific T-cells. Representative density plots illustrating the expression and frequency of IFNγ⁺ and TNFα⁺ producing cells are shown as percentage within the CD154⁺ cells from healthy non-relatives (NR), first-degree relatives (FDR), Crohn´s disease (CD), Ulcerative colitis (UC), active or in remission (-R),celiac disease patients (CeD) ± gluten-free diet (GFD), refractory (Refr) patients and rheumatoid arthritis patients (RA) are shown ± gliadin stimulation.
